# Soil bacterial and fungal communities respond differently to various isothiocyanates added for biofumigation

**DOI:** 10.3389/fmicb.2014.00729

**Published:** 2015-01-07

**Authors:** Ping Hu, Emily B. Hollister, Anilkumar C. Somenahally, Frank M. Hons, Terry J. Gentry

**Affiliations:** ^1^Department of Soil and Crop Sciences, Texas A&M UniversityCollege Station, TX, USA; ^2^State Key Laboratory of Forest and Soil Ecology, Institute of Applied Ecology, Chinese Academy of SciencesShenyang, China

**Keywords:** seed meal, isothiocyanates, soil microbial community, pyrosequencing

## Abstract

The meals from many oilseed crops have potential for biofumigation due to their release of biocidal compounds such as isothiocyanates (ITCs). Various ITCs are known to inhibit numerous pathogens; however, much less is known about how the soil microbial community responds to the different types of ITCs released from oilseed meals (SMs). To simulate applying ITC-releasing SMs to soil, we amended soil with 1% flax SM (contains no biocidal chemicals) along with four types of ITCs (allyl, butyl, phenyl, and benzyl ITC) in order to determine their effects on soil fungal and bacterial communities in a replicated microcosm study. Microbial communities were analyzed based on the ITS region for fungi and 16S rRNA gene for bacteria using qPCR and tag-pyrosequencing with 454 GS FLX titanium technology. A dramatic decrease in fungal populations (~85% reduction) was observed after allyl ITC addition. Fungal community compositions also shifted following ITC amendments (e.g., *Humicola* increased in allyl and *Mortierella* in butyl ITC amendments). Bacterial populations were less impacted by ITCs, although there was a transient increase in the proportion of *Firmicutes*, related to bacteria know to be antagonistic to plant pathogens, following amendment with allyl ITC. Our results indicate that the type of ITC released from SMs can result in differential impacts on soil microorganisms. This information will aid selection and breeding of plants for biofumigation-based control of soil-borne pathogens while minimizing the impacts on non-target microorganisms.

## Introduction

There is increasing demand for food grown organically, using alternative pest control practices such as biofumigation, due to the concerns about the toxicity of commercial pesticides. The most well-studied biofumigation system involves plants from the family *Brassicaceae* which produce glucosinolates (GLS) that, once incorporated into soil, hydrolyze into a variety of biocidal products including isothiocyanates (ITCs), nitriles, organic thiocyanates, SCN^−^, oxazolidinethione, and epthionitriles (Cole, 1976; Borek and Morra, 2005). The ITCs have received particular attention since they strongly inhibit a variety of soilborne plant pathogens including *Rhizoctonia* spp. (Cole, 1976), *Aphanomyces euteiches* f. sp. *pisi* (Smolinska et al., 1997), and *Phymatotrichopsis omnivora* (Duggar) Hennebert (Hu et al., 2011). Several studies have also demonstrated that the inhibitory effects of ITCs can vary dramatically for different microorganisms and with the type of ITC added (Kirkegaard et al., 1996; Bending and Lincoln, 2000; Manici et al., 2000; Hu et al., 2011; Somenahally et al., 2011).

The majority of these studies were conducted on pure cultures of isolated organisms instead of organisms within their natural environment, e.g., soil. The impacts of the various ITCs may be very different within the soil environment due to complex interactions with soil solids and the phase-partitioning of ITCs between soil phases (Borek et al., 1998; Matthiessen and Shackleton, 2005). Moreover, studies adding ITCs in a pure chemical form without also adding decomposable plant tissue would not resemble real-world biofumigation strategies where the ITCs would be added within plant biomass (e.g., oilseed meals—the residue remaining after extraction of oil). These studies adding only pure ITCs may detect direct impact of the ITCs on target populations but would miss any indirect effects due to changes in overall soil microbial activity, abundance, and community composition that normally occur during decomposition of organic residues (Baldrian et al., 2011; Hollister et al., 2012). These changes in the non-target microbial community could further impact the efficacy of the biofumigation process by either enhancing or inhibiting microbial populations capable of suppressing plant pathogens through competition or antagonistic interactions. Furthermore, changes in non-target populations could potentially impact ecosystem functions and health by altering important soil biogeochemical processes such as C cycling (Troncoso-Rojas et al., 2009).

Of the few studies that have investigated the impacts of ITCs on microbial communities within soil, most have been focused exclusively upon bacteria (Bending and Lincoln, 2000; Ibekwe et al., 2001). The handful of studies that have investigated the impacts of ITCs on soil fungal composition have used low-resolution techniques such as DGGE and fatty acid methyl ester analysis which provided information regarding community shifts but little-to-no information regarding which specific organisms were being impacted (Rumberger and Marschner, 2003; Troncoso-Rojas et al., 2009). Hollister et al. (2012) were the first to use high-throughput sequencing methods to characterize the impact of *Brassica juncea* oilseed meals (SM; releasing allyl ITC) on soil fungal and bacterial communities. They found that the *B. juncea* SM had a dramatic impact upon the composition of both the fungal and bacterial communities and resulted in a >60% reduction in fungal diversity and enrichment with bacterial taxa rich in strains associated with fungal disease suppression (e.g, *Bacillus*, *Pseudomonas*, *Streptomyces*).

In order to test the impacts of other ITCs on soil microbial communities in the presence of accompanying plant biomass, we conducted a study by applying various pure ITCs to soil along with flax SM (chemically similar to other SMs but releasing no biocidal compounds such as ITCs), and determined the resulting impacts on soil fungal and bacterial communities. This approach allowed us to more accurately determine the sole impacts of the different ITCs since the exact same SM was added to all treatments, as opposed to adding SMs from different plants that naturally varied in their ITC content but may have also varied in other chemical properties that would have confounded interpretation of the results (Osono et al., 2003; Omirou et al., 2011). To be more explicit, the objective is to investigate ITC effects that simulate closely their application in practice (together with oilseed meal that would in agricultural practice release them) instead of exploring pure ITC impacts on soil microbial communities.

## Materials and methods

### Soil and oilseed meal

Weswood loam soil (fine-silty, mixed, superactive, thermic, Udifluventic Haplustept) was collected from the Texas A&M AgriLife Research Farm near College Station, TX Weswood soils are well drained loamy soils generally containing low levels of nutrients and organic matter and are used as irrigated cropland (USDA NRCS, 2008). Bulk soil samples were collected from 0 to 15 cm depth and then homogenized and passed through a 2-mm sieve. The soil water content was then determined by oven-drying a subsample of 20 g of field moist soil for 24 h at 105°C and calculated to be 14.4% (w/w). Soil samples were incubated at room temperature (~24°C) for 24 h before use. Soil characteristics were tested as described by Hu et al. (2011), and the testing results were summarized in Table S1.

Flax (*Linum usitatissimum* L.) oilseed meal was obtained by processing seeds with a Komet Oil Press (Model CA59, IBG Monforts Oekotec, Germany). The resulting flax SM was ground with a mortar and pestle and passed through a 1-mm sieve. The water content of the SM was determined by drying sub-samples at 60°C for 3 days. Chemical composition and glucosinolate (GLS) concentration of flax SM were determined as described by Hu et al. (2011) and are summarized in Table S2.

### Microcosm setup

This was a laboratory microcosm study investigating soil treated with different types of ITCs, including allyl (2-Propenyl) ITC (Acros Organics, Fair Lawn, NJ, USA), butyl ITC (Alfa Aesar, Ward Hill, MA, USA), phenyl ITC (MP Biomedicals, Solon, Ohio, USA), and benzyl ITC (Acros Organics, Fair Lawn, NJ, USA). The choice of these ITCs in our study was based on their precursor glucosinolate presence in *Brassicaceous* family, their representation of aliphatic and aromatic ITCs, and their use in previous studies. Soil was amended with ITCs to achieve a concentration of 50 μg ITC g^−1^ soil, which is comparable with allyl ITC levels in previous biofumigation studies (Charron and Sams, 1999; Hu et al., 2011). Each treatment had three replications, and there were three controls receiving no ITC (only sterile water added). The microcosms were set up in 130-cm^3^ sterile specimen containers (VWR International, LLC., Sugar Land, TX, USA) filled with 57.2 g (50 g dry soil equivalent) fresh soil. A total of 0.52 g (0.5 g dry SM equivalent) flax SM was then mixed with soil in each of the microcosm including the three controls. Each ITC (2.5 mg) was individually mixed with 3.0 ml sterile water and vortexed for 1 min to homogenize before adding to the microcosms to generate an initial ITC concentration of 50 μg g^−1^ soil. The lids on the microcosms were left loose in order to maintain aerobic conditions. The microcosms were incubated at 25°C for 28 days. Soil subsamples (2 g) were collected at days 2, 7, 14, 21, and 28 and stored at −80°C until DNA extraction. Soil moisture was adjusted to 14.4% every 24 h by addition of sterile water.

### DNA extraction and quantification

Community DNA was extracted from 0.5 g aliquots of each soil sample using a PowerSoil DNA extraction kit (Mo Bio Laboratories, Inc., Carlsbad, CA, USA). Extracted DNA was purified with illustra MicroSpin S-400 HR columns (GE Healthcare Bio-Sciences Corp, Piscataway, NJ, USA) and quantified using a Quant-iT PicoGreen dsDNA assay kit (Invitrogen Corp, Carlsbad, CA, USA).

### qPCR on general bacteria and fungi

Community qPCR assays, based upon Fierer et al. (2005) and Boyle et al. (2008) were used to evaluate the relative abundances of general bacteria and fungi in the microcosm communities. Assays were performed in triplicate, using a Rotor-Gene 6000 series thermal cycler (Qiagen, Valencia, CA, USA). For general bacterial and fungal qPCR, each 15 μL reaction contained: 6.75 μL 2.5x RealMasterMix with 20x SYBR solution (5Prime, Inc., Gaithersburg, MD, USA), 1.5 μL BSA (10 mg mL^−1^), 0.75 μL of each primer (10 μM), 0.25 μL molecular-grade water, and 5.0 μL template DNA (1.0 ng μL^−1^). Thermocycling consisted of an initial denaturation at 95°C for 15 min, followed by 40 cycles of 95°C for 1 min and annealing temperature at 53°C for 30 s, and 72°C for 1 min. Primer sets of Eub338/518 (Fierer et al., 2005) and 5.8S/ ITS1F (Boyle et al., 2008) were used for bacteria and fungi respectively. Plasmid standards for the bacterial and fungal relative abundance by qPCR were generated as described by Somenahally et al. (2011). Briefly, we used *Escherichia coli* DH10B (pUC19) and *Neurospora crassa* as the source for standards. After PCR, the amplicons were confirmed on agarose gel and cloned into a pGEM®-T Easy vector (Promega, Madison, WI, USA). Then the positive clones were isolated and extracted with Wizard SV Miniprep kit (Promega, Madison, WI, USA). Melting curve analysis was conducted to verify amplification of the correct product.

### Fungal and bacterial tag-encoded amplicon pyrosequencing and analysis

Purified community DNA samples were submitted to the Research and Testing Laboratory (Lubbock, TX, USA) for tag-pyrosequencing using 454 GS FLX titanium technology (454 Life Sciences, Branford, CT, USA). The fungal ITS region was amplified using primers ITS1F and ITS4 for the initial generation of the amplicons (Amend et al., 2010), and fungal amplicons were sequenced in the forward direction, generating reads from ITS1F. Bacterial 16S rRNA genes were sequenced in a similar manner as the fungal sequences substituting primers 530F and 1100R as described by Acosta-Martínez et al. (2008) to generate initial amplicons. Bacterial amplicons were also sequenced in the forward direction.

Fungal sequences were preprocessed in MOTHUR v.1.20.0 (Schloss et al., 2009) to remove primers and barcodes, check quality (Q25), discard sequences that contain ambiguous base calls, cap the homopolymer length at 8, and remove sequences that were shorter than 300 bp in length. Chimeric sequences were then identified from the ITS sequence libraries using the Fungal Metagenomics Pipeline chimera tool (http://www.borealfungi.uaf.edu) provided by the University of Alaska Fairbanks. All potentially chimeric reads were flagged and excluded from downstream analysis. Sequences from all samples were combined in one single file and clustered into OTUs (97% similarity) using CD-HIT-EST (Li and Godzik, 2006). Identities were assigned to the OTUs using the UNITE database's 454 pipeline (Troncoso-Rojas et al., 2009) by submitting representative sequences for BLAST. Hits with BLAST scores ≤200 or query percentage of alignment ≤60% were considered to represent unknown or unclassified fungi. Rarefaction curves based upon the OTU data were calculated in MOTHUR v.1.20.0 (Schloss et al., 2009).

Bacterial sequence processing was carried out as described by Schloss et al. (2011). Initial sequences were all preprocessed in MOTHUR v.1.22.0 (Schloss et al., 2009) to remove primers and barcodes, check quality (Q25), discard sequences that contained ambiguous base calls, cap the homopolymer length at 8, remove sequences that were shorter than 250 bp in length. Resulting sequence data were then aligned, and chimera checked with the chimera.uchime function. All sequences that were flagged as potential chimeras were excluded from downstream analysis.

All tag pyrosequence data from this study were deposited and made public accessible in the MG-RAST under accession numbers 4515099.3 (ITS reads) and 4515300.3 (16S reads).

### Statistical analysis

Variation in community qPCR values among amendment types and over time were assessed using SAS version 9.2 (SAS Institute Inc., 2003). Proc GLM was used to test individual treatment significance. Pair-wise treatment mean comparisons were made using Least Significance Difference (LSD) when treatment was shown to be significant. Unless otherwise indicated, all statistical significance levels were set as *P* ≤ 0.05. Values were log-transformed prior to analysis.

Nonmetric multidimensional scaling (NMDS) of the bacterial and fungal communities based upon OTU composition was carried out using the Bray-Curtis similarity metric in the PAST software package, version 2.03 (Hammer et al., 2001). Two-Way analysis of similarity (ANOSIM) on soil fungal and bacterial OTU profiles with respect to the effects of ITC type and incubation time were conducted in PAST. Samples were clustered using Unweighted Pair Group Method with Arithmetic mean (UPGMA) based on Bray-curtis distance matrix in QIIME 1.8.0 (Caporaso et al., 2010). All above analyses were based on sub-sampled OTU counts across all samples with even number of sequences (1036 for fungi and 1558 for bacteria).

## Results and discussion

### Abundance of soil fungal and bacterial populations

Since our experiment design aimed to simulate various ITC releasing oilseed meal application to soil in agricultural practice, all results on soil microbial community we will be discussing here should be attributed to a combined effect of ITCs and flax oilseed meal addition instead of pure ITC effects alone.

**Figure 1:**
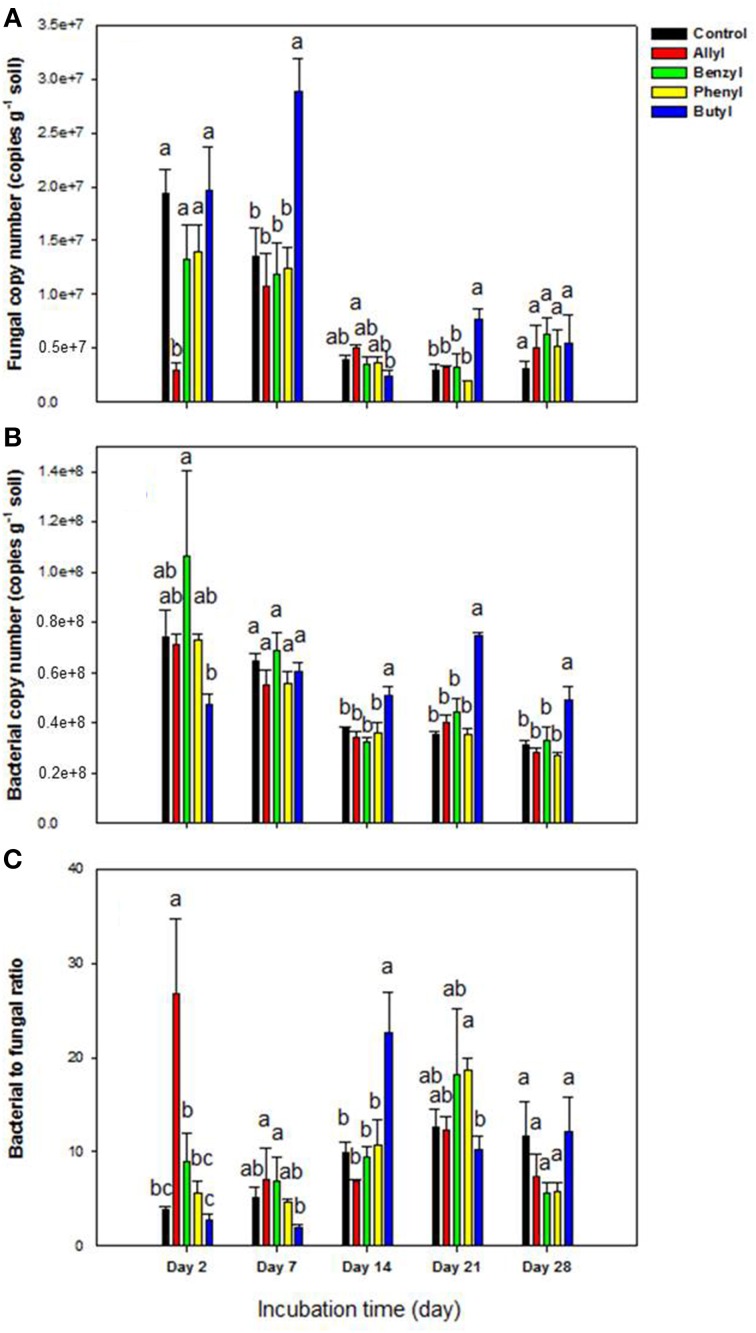
**Microbial abundance by qPCR in Weswood loam soil 2, 7, 14, 21, and 28 days after amendment with 1% flax SM and 50 μg g^−1^ allyl, benzyl, butyl or phenyl isothiocyanate (ITC)**. The control received 1% flax SM but no ITC. Bars represent the mean of 3 biological replicates for each treatment, and error bars represent standard deviation. **(A)** Soil fungal copy number. **(B)** Soil bacterial copy number. **(C)** The ratio of soli bacterial to fungal copy number. Different letters indicate significant difference at *P* < 0.05 within each day.

Of the 4 types of ITCs used in our study, fungal numbers were only significantly impacted by the two aliphatic (allyl and butyl) ITCs at several sampling time points (Figure 1). To be specific, these two ITCs had an opposite effect with the allyl ITC significantly suppressing fungal abundance after 2 days of incubation. In contrast, butyl ITC resulted in significantly higher fungal levels after 7 days compared to the other ITC-treated soils and the unamended control (Figure 1A). In general, soil bacterial numbers were not impacted as much by the ITCs as fungi were (Figure 1B). Butyl ITC appeared to initially suppress bacterial numbers and resulted in significantly higher bacterial numbers from 14 to 28 days. The bacteria: fungi ratio was similar to the above results. To be specific, the allyl ITC significantly increased the ratio by 2 days, and the butyl ITC had a significantly higher ratio than all other treatments at 14 days (Figure 1C). By 28 days, the only significant difference among the treatments was the slightly higher bacterial levels in the butyl ITC-amended soil.

As other studies have shown, the incorporation of ITCs temporarily inhibited soil fungi with varied suppression levels according to the ITC type (Yulianti et al., 2007; Hu et al., 2011), with allyl ITC having a greater inhibitory effect on the overall soil fungal population size than did the aromatic ITCs (benzyl and phenyl) (Angus et al., 1994; Matthiessen and Shackleton, 2005; Troncoso-Rojas et al., 2009; Hu et al., 2011). Interestingly, our results also indicated that butyl ITC may be more suppressive to soil bacterial populations than the other ITCs. The decrease in bacterial populations was followed by fungal proliferation, which suggested that addition of butyl ITC indirectly increased fungal populations through less competition from the reduced bacterial community.

The higher level of inhibition by aliphatic relative to aromatic ITCs in our study may be due to higher chemical volatility and/ or higher biological activity of aliphatic ITCs, although it can be difficult to predict bioavailability and toxicity in soil due to complex interactions with the soil matrix (Borek et al., 1998; Matthiessen and Shackleton, 2005).

### Soil fungal community composition

**Figure 2:**
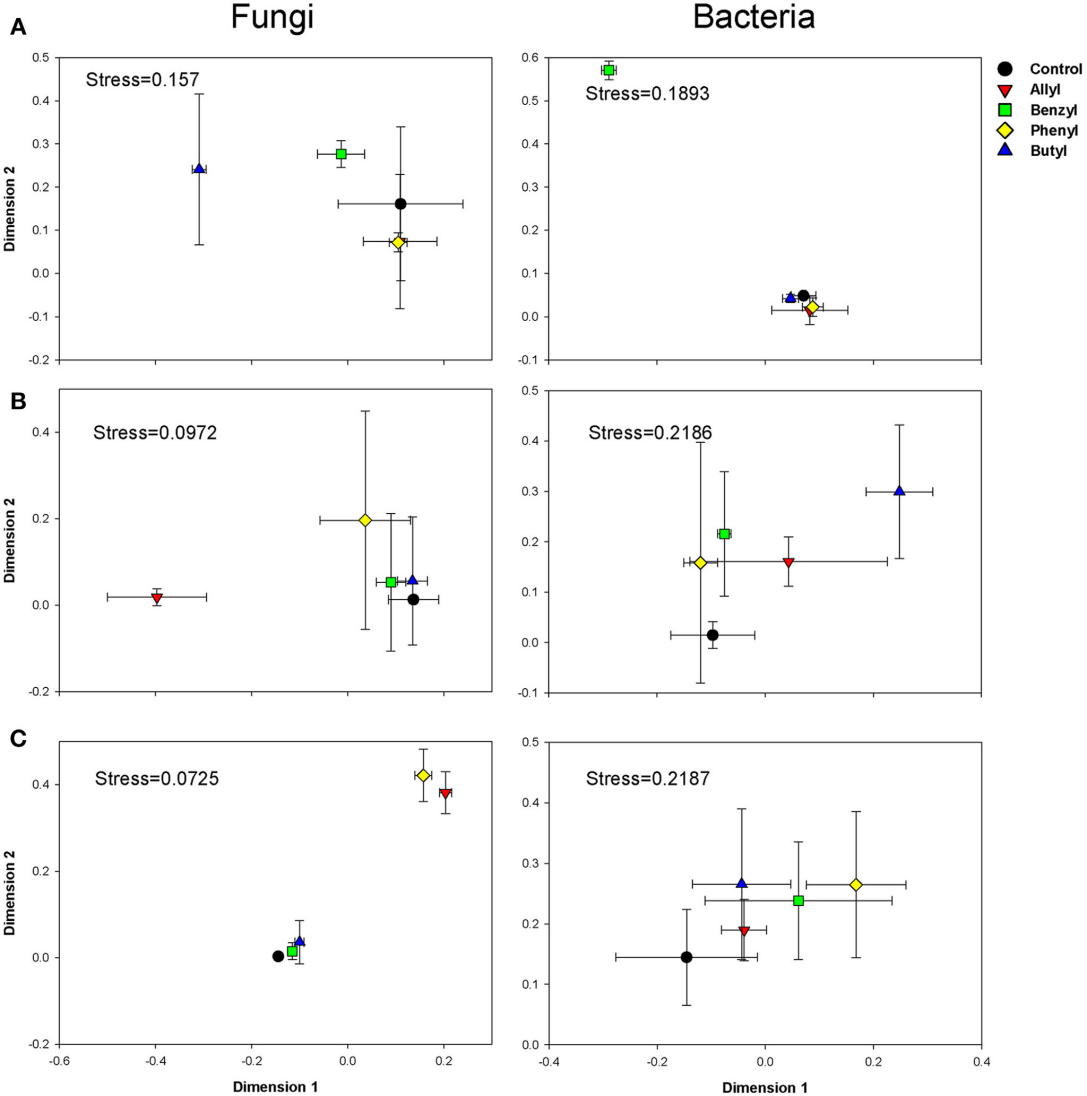
**NMDS graphs of fungal and bacterial communities in Weswood loam soil at 2 (A), 7 (B), and 28 (C) days after amendment with 1% flax SM and 50 μg g^−1^ allyl, benzyl, butyl or phenyl isothiocyanate (ITC)**. All sequences were deposited in MG-RAST with accession number of 4515099.3 for fungal ITS and 4515300.3 for 16S. Analysis was carried out based on sub-sampled operational taxonomic units (OTUs) clustered at 97% sequence identities with even number of sequences (1036 for fungal and 1588 for bacteria). The control received 1% flax SM but no ITC. Symbols represent the mean of 3 biological replicates for each treatment, and error bars represent standard deviation.

Soil fungal community compositions based on OTU profiles were significantly different with respect to ITC type and time of incubation, as indicated by Two-Way ANOSIM analysis (Table S3). The NMDS analysis indicated that amendment of soil with various ITCs altered the soil fungal community composition (Figure 2). At day 2, when the soil fungal population levels were greatly inhibited by allyl ITC, the composition of the fungal community in that treatment was surprisingly similar to the control. In contrast, the butyl ITC treatment, which did not suppress soil fungal numbers, did change the composition of the soil fungal community. By 7 days, the allyl ITC had resulted in a dramatic shift in the soil fungal community composition. These differences in the allyl ITC treatments persisted through 28 days. At the end of the incubation, the fungal community composition in all of the ITC amendments remained different from the control, with allyl and phenyl being more different than butyl and benzyl amendments.

**Figure 3:**
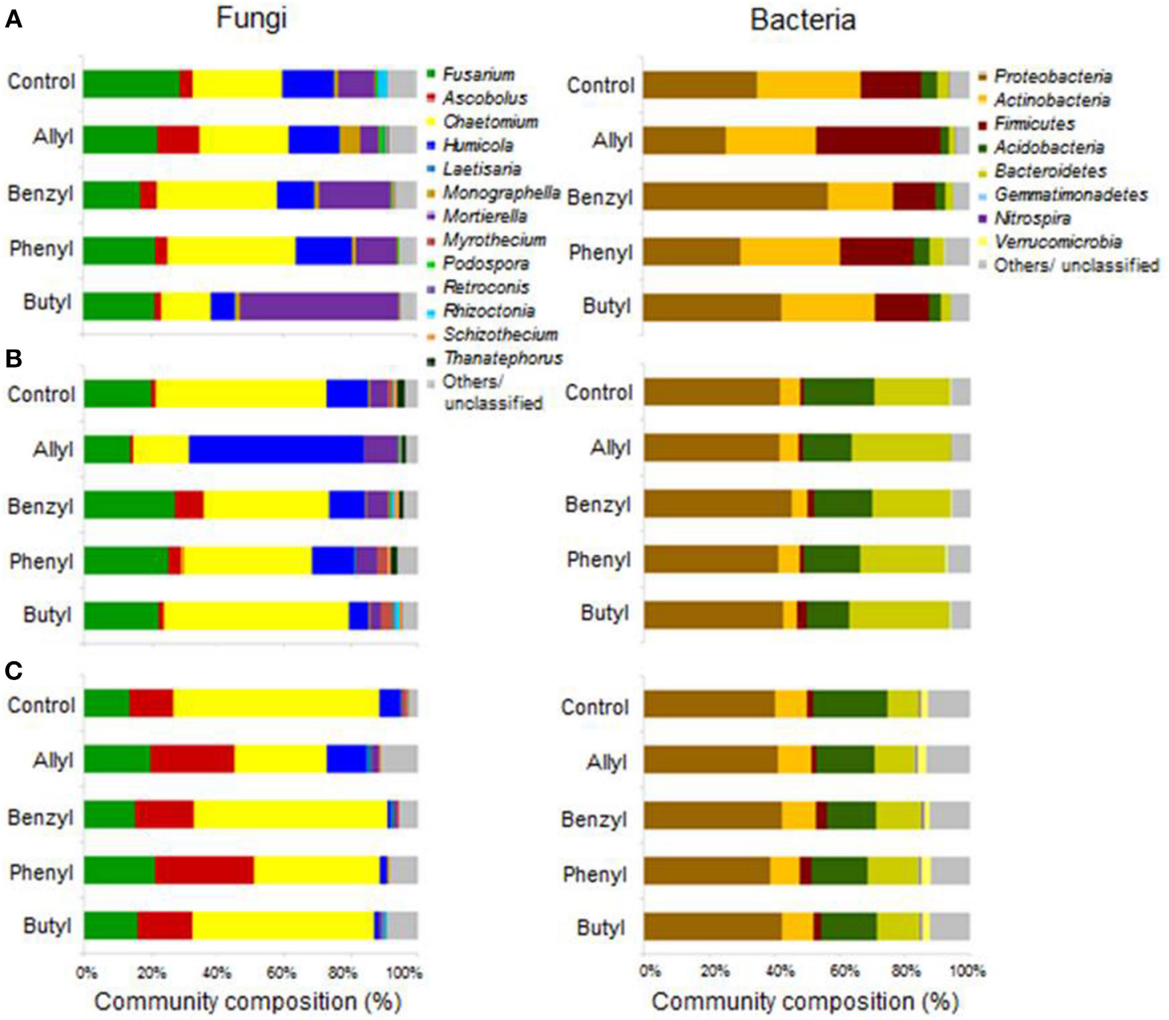
**Soil microbial operational taxonomic unit (OTU) distribution patterns in a Weswood loam at 2 (A), 7 (B), and 28 (C) days after amendment with 1.0% flax SM and 50 μg g^−1^ allyl, benzyl, phenyl or butyl isothiocyanate (ITC)**. The controls received 1% flax SM but no ITC. Bars represent the mean of 3 biological replicates for each treatment. All sequences were deposited in MG-RAST with accession number of 45150099.3 for fungal ITS and 4515300.3 for 16S.

Soil fungal taxonomic distribution patterns were significantly different with respect to ITC type and time of incubation, as indicated by Two-Way ANOSIM analysis (Table S4), although fungal identifications were just nearest neighbors in a partial database, identified by BLAST (Figure 3). *Ascomycota* and *Mortierellomycotina* were the dominant most closely related phylum and subphylum of classified fungi in all treatments (89–97%). *Fusarium*, *Chaetomium*, *Humicola*, *Mortierella*, and *Ascobolus* were the dominant most closely related genera detected in all treatments as well as the control through time (Table 1). Among those, the fungal genera that responded the most to ITC amendments were most closely related to *Chaetomium*, *Humicola*, and *Mortierella*. At 2 days, the butyl ITC treatment yielded significantly lower relative abundance of *Chaetomium* and *Humicola*, and a significantly higher relative abundance of *Mortierella*, both of which contributed to its unique fungal taxonomic distribution in comparison to the other treatments. Later at 7 days, allyl ITC application yielded significantly suppressed *Chaetomium* but enhanced *Humicola* compared with the control and the other ITC treatments (Figure 3). After 28 days, *Chaetomium* were the dominant fungi (28–62%) and *Mortierella* had decreased to a minor component (<2%) of fungal communities in all treatments. However, even after 28 days, the allyl ITC treatment contained a significantly lower proportion of *Chaetomium* and more *Humicola* than most of the other treatments did.

**Table 1:**
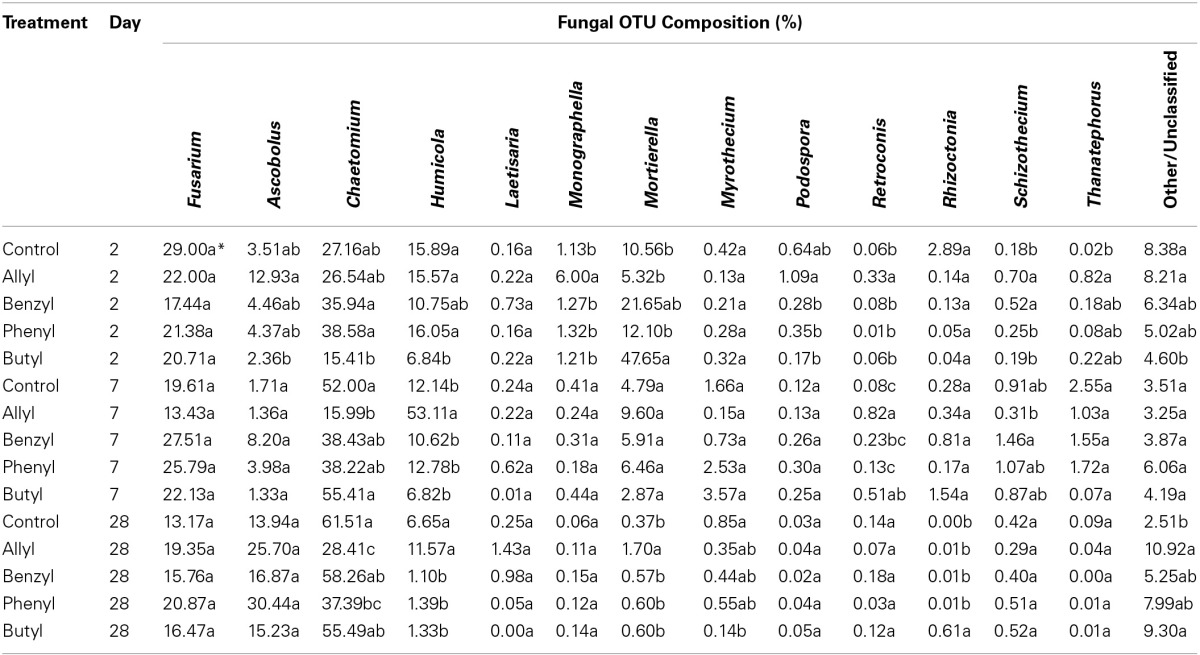
**Fungal operational taxonomic unit (OTU) composition summarized at the genus level in a Weswood loam soil mixed with 1% flax SM and treated with 50 μg g^−1^ allyl, benzyl, phenyl, or butyl isothiocyanate (ITC) and the control receiving no ITC after 2, 7, and 28 days of incubation at 25°C**.

*Values displayed represent the mean of 3 biological replicates for each treatment. All sequences were deposited in MG-RAST with accession number of 4515099.3 for fungal ITS*.

^*^Different letters indicate significant difference at P < 0.05 within each time point for each genus.

These results are the first to detail the impacts of various ITCs on soil fungal community composition, so there is no direct point-of-comparison in the published literature. A related study by Hollister et al. (2012) partially supported our findings that soil amendment of ITC-releasing SM did alter soil fungal community structure compared with non-ITC-producing SM within only 3 days of incubation. Our study also suggested that fungal re-colonization in the soil may result in differentiation due to the varied initial ITC suppressive impacts, especially in the case of allyl ITC. Aromatic ITCs also shifted soil fungal community structure given a longer incubation time of 4 weeks, which was likely due to systematic impacts instead of pure chemical effects, considering that ITCs typically degrade rapidly (within hours to days) when incorporated into soil (Warton et al., 2003; Gimsing and Kirkegaard, 2008).

In terms of taxonomic classification of the soil fungal communities, *Ascomycota* as the largest group of the true fungi (Larena et al., 1999) that was not surprisingly found likely to be dominant. The other possible dominating fungal group *Mortierellomycotina* includes fast-growing members that utilize substrates that are high in sugar and are considered able to degrade plant materials (Kirk et al., 2001; James and O'Donnell, 2007).

Several of the fungi dominated our soil fungal community was most closely related to the genus *Mortierella* within the subphylum *Mortierellomycotina*. *Mortierella* spp. has been reported to be tolerant of several fungicides and be capable of surviving soil fumigation and then rapidly re-colonize the soil (Warcup, 1976; Kuthubutheen and Pugh, 1979). Since we observed a dramatic proliferation of this fungal group in the butyl ITC treatment, it is likely that *Mortierella* possessed higher tolerance to butyl ITC relative to the other ITCs in our study.

Among the *Ascomycota* groups found in our soils, *Humicola* and *Chaetomium* were likely the ones that most dramatically responded to various ITC types. Allyl ITC-treated soil was rapidly re-colonized, primarily by *Humicola*, which are considered to be beneficial soil fungi with some representatives having been used to produce important enzymes for hydrolyzing lignocellulosic materials in the renewable energy industry (Lang et al., 2011). The ability of *Humicola* to re-colonize soil rapidly has also been reported in previous research after suppression by fungicides (Kuthubutheen and Pugh, 1979). It could therefore partially explain our finding that *Humicola* out-competed other genera such as *Chaetomium* in the allyl ITC treatment. Interestingly, none of the ITC treatments decreased the percentage of general *Fusarium* spp. in the fungal communities; however, this could actually be beneficial for controlling specific strains of *Fusarium*-related pathogens (e.g., *Fusarium oxysporum*), since most *Fusarium* spp. are non-pathogenic and some are even antagonistic against pathogenic strains and can induce systemically acquired resistance in plants (Abadie et al., 1998).

### Soil bacterial community composition

Soil bacterial community compositions based on OTU profiles were found to be significantly different with respect to ITC type and time of incubation, as indicated by Two-Way ANOSIM analysis (Table S3). NMDS analysis indicated that the impacts of ITC applications on soil bacterial community composition was less-pronounced than they were for the fungi (Figure 2). Of the two aliphatic ITCs, allyl, and butyl ITC shifted the soilbacterial composition compared with the control only at day 7. The aromatic ITCs, benzyl ITC had a transient effect and altered soil bacterial community compositions after 2 days of incubation, but these differences were diminished by 28 days. Phenyl ITC shifted soil bacterial community structure after 4 weeks, which was likely due to systematic impacts rather than pure chemical effects.

Distributions of *Firmicutes* were only significantly different with respect to time of incubation, as indicated by Two-Way ANOSIM analysis (Table S4). Soil bacterial taxonomic distribution patterns were only transiently shifted due to ITC applications (Figure 3). The dominant bacterial phyla detected were *Proteobacteria*, *Actinobacteria*, *Firmicutes*, *Acidobacteria*, and *Bacteroidetes*. The only bacterial phylum that differentially responded to ITC addition (compared to the control) was *Firmicutes* at 2 days, which consisted of 4 dominant genera detected in our microcosms including *Bacillus*, *Brevibacillus*, *Lysinibacillus*, and *Paenibacillus* (Table 2). Allyl ITC significantly increased the proportion of *Firmicutes* (38%), mainly *Brevibacillus* and *Paenibacillus*, compared to the control and all of the other ITC treatments (12–19% *Firmicutes*). However, after 7 days, *Firmicutes* was a less dominant component (<4%) of bacterial communities in all treatments being replaced largely by *Bacteroidetes* and *Acidobacteria*; bacterial taxonomic profiles in all treatments became similar to each other at phylum level by 28 days.

**Table 2:** **Bacterial operational taxonomic unit (OTU) composition summarized at the genus level for *Firmicutes* in a Weswood loam soil mixed with 1% flax SM and treated with 50 μg g^−1^ allyl, benzyl, phenyl, or butyl isothiocyanate (ITC) and the control receiving no ITC after 2, 7, and 28 days of incubation at 25°C**.

**Treatment**	**Day**	**Total *Firmicutes***	***Firmicutes* OTU Distribution (% of Bacterial Community)**				
			***Bacillus***	***Brevibacillus***	***Lysinibacillus***	***Paenibacillus***	**Others/Unclassified**
Control	2	18.46b^*^	7.28a	0.74ab	0.01b	2.99b	7.44a
Allyl	2	38.00a	8.92a	6.48a	0.67a	14.15a	7.77a
Benzyl	2	12.80b	5.03a	0.29b	0.00b	1.97b	5.51a
Phenyl	2	22.58b	8.65a	1.92ab	0.00b	4.05b	7.95a
Butyl	2	16.61b	5.89a	0.52b	0.00b	4.85b	5.35a
Control	7	0.96a	0.41a	0.00a	0.00a	0.05b	0.50a
Allyl	7	1.32a	0.70a	0.00a	0.00a	0.14ab	0.48a
Benzyl	7	1.93a	0.87a	0.00a	0.00a	0.08b	0.98a
Phenyl	7	1.19a	0.42a	0.00a	0.00a	0.08b	0.69a
Butyl	7	3.02a	1.37a	0.01a	0.00a	0.22a	1.42a
Control	28	1.86a	0.90a	0.03a	0.00a	0.08a	0.85a
Allyl	28	1.48a	0.78a	0.00a	0.00a	0.06a	0.64a
Benzyl	28	3.39a	1.70a	0.00a	0.00a	0.12a	1.57a
Phenyl	28	3.54a	1.78a	0.02a	0.00a	0.19a	1.55a
Butyl	28	2.42a	1.25a	0.01a	0.00a	0.09a	1.06a

*Values displayed represent the mean of 3 biological replicates for each treatment. All sequences were deposited in MG-RAST with accession number of 4515300.3 for 16S*.

* *Different letters indicate significant difference at P < 0.05 within each time point for each phylum or genus*.

It is especially interesting that allyl ITC selectively impacted the soil bacterial communityin terms of taxonomic composition. A significant difference was detected in the early stages with allyl ITC increasing the proportion of *Firmicutes* compared to the other three ITCs. It was likely that this group of bacteria was more resistant to allyl ITC toxicity than the other bacterial members were. When further examined at the genus level, we found that the most dominant *Firmicutes* were most similar to *Paenibacillus*, which includes members that are known to be tolerant to pesticides (Singh et al., 2009) and also suppressive to soil-borne fungal pathogens (notably *Fusarium* and *Chaetomium* spp.) through various mechanisms such as chitinase production (Budi et al., 2000; Guemouri-Athmani et al., 2000; Da Mota et al., 2005; Singh et al., 2009). Since studies, including ours, generally report that allyl ITC is among the most effective ITCs for biofumigation against fungal populations, this suggests the possibility that bacterial competition due to increased populations of antagonistic bacteria contributed to the observed suppression of fungi following biofumigation with allyl ITC. Although direct toxicity of allyl ITC against fungi is likely the primary mechanism responsible for short-term inhibition, it is likely that complex biological interactions and competition play a role in the continued suppression of fungal pathogens. It should be pointed out that the specific impacts on bacterial and fungal taxonomic (lower level) composition observed in this study were likely the result of multiple factors such as ITC level, nutrient amount, soil water content, the way the ITC was incorporated, and the soil tested, each of which alone has been reported to greatly affect microbial responses to amendments in previous studies (Borek et al., 1998; Smith and Kirkegaard, 2002; Rumberger and Marschner, 2003; Matthiessen and Shackleton, 2005; Hu et al., 2011; Hollister et al., 2012; Wang et al., 2012). For example, if the ITCs were added to the flax SM first (before incorporation into soil) instead of adding them to the soil-SM mixture, this might have affected the ITC bioavailability and produced different results. Additional studies are needed to further elucidate the role that the altered bacterial and fungal communities, following biofumigation, may play in longer-term suppression of fungal pathogens. This information will be very useful for producers designing biofumigation strategies for pathogen control, for plant breeders selecting plants for controlling specific pathogens, and for ecologists attempting to determine the effects of land-applied ITC-releasing SMs on soil quality.

## Conclusions

This study provided the most comprehensive characterization of the differing impacts of various isothiocyanates on soil bacterial and fungal communities. Our findings are the first to detail the impacts of various ITCs, in the presence of flax SM application, on soil fungal and bacterial community composition. By using pure ITCs and a single type of SM, it enabled us to focus specifically upon differential effects of the ITCs and eliminate other variables such as varying chemical composition (C, N, S, other biocidal chemicals, etc.) inherent in comparisons of ITC-producing (e.g., mustard) and non-ITC-producing (e.g., flax) SMs in a direct way. The application of allyl ITC in the presence of flax SM temporarily decreased soil fungal populations. Conversely, butyl ITC decreased bacterial populations, and the other ITCs did not significantly impact bacterial or fungal population levels. Soil fungal communities seemed to be more sensitive to aliphatic (allyl and butyl) than to aromatic (benzyl and phenyl) ITCs, while soil bacterial communities were impacted by both types of ITCs. Allyl and butyl ITCs had a wide-spectrum initial inhibiting effect on soil fungal or bacterial communities, respectively. On the other hand, the selectivity of these ITCs was also apparent among several fungal and bacterial genera with ITC amendment leading to microbial community composition changes. Certain microbial community composition changes observed showed increased proportions in bacterial taxa which include bacteria associated with fungal disease suppression. The increase in these bacteria and decrease in overall fungal populations following amendment with allyl ITC suggests that the observed efficacy of allyl ITC on fungal suppression was not only due to direct toxicity of allyl ITC against soil fungi but also to biological interactions and competition with the altered microbial community that existed following fumigation. However, the importance and efficacy of such community changes was not tested in this work and needs further investigation. The robustness of the microbial community shifts observed in our study should be further validated in contrasting soils differencing in characteristics known to affect microbial community composition and structure, such as pH and organic matter quality. Although there are limitations in this study, our results would provide a strong foundation and justification for subsequent studies investigating how different soil types, vegetation history, and climate etc., interact to affect the isothiocyanate impacts on soil microbial communities.

### Conflict of interest statement

The authors declare that the research was conducted in the absence of any commercial or financial relationships that could be construed as a potential conflict of interest.
