# Supplementary material for: Soil bacterial and fungal communities respond differently to various isothiocyanates added for biofumigation
Source: Front Microbiol. 2015 Jan 7;5:729. doi: 10.3389/fmicb.2014.00729 (PMC4288022; doi:10.3389/fmicb.2014.00729)
Supplement: Supplementary file 1 [file DataSheet1.DOCX]

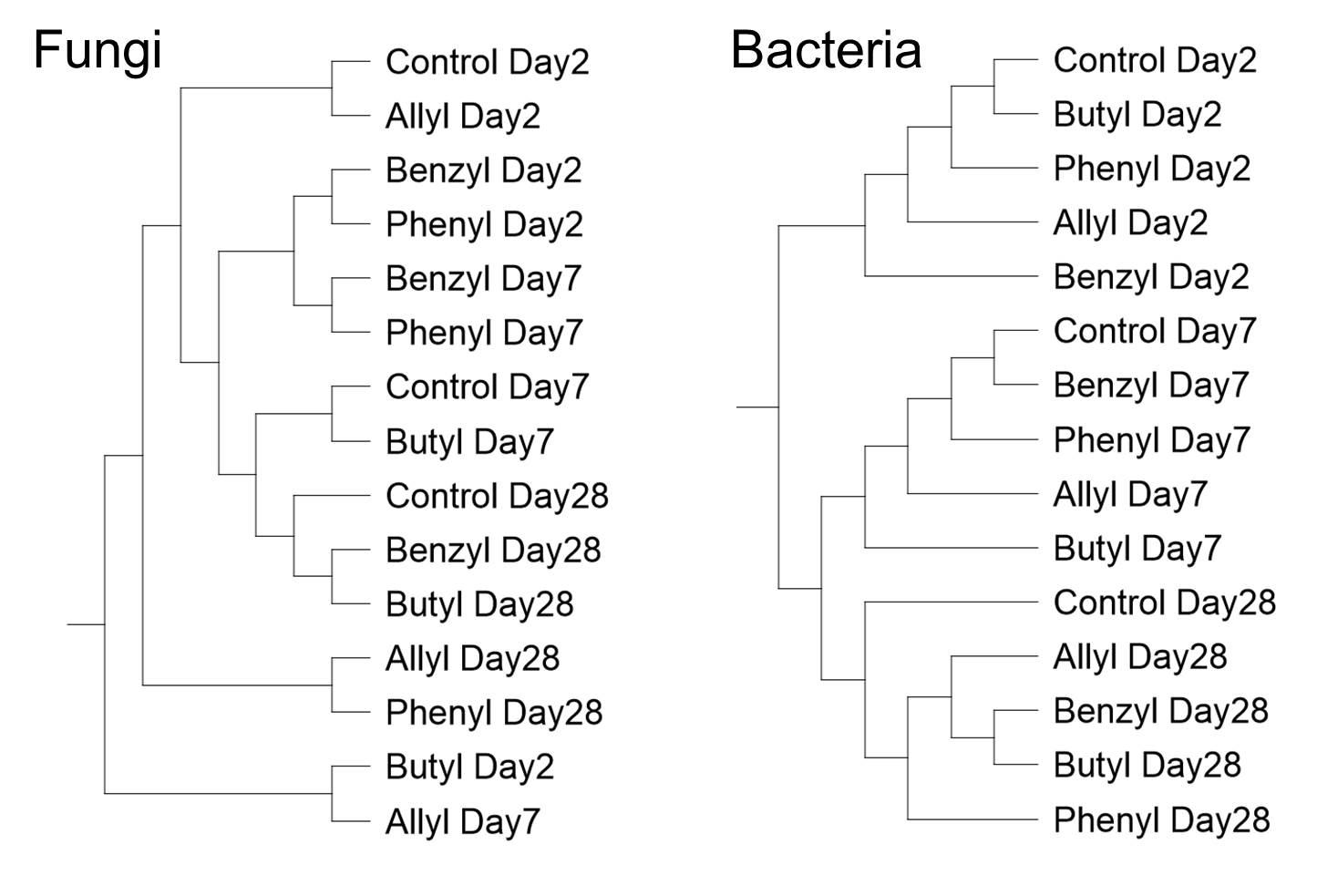


Fig. S1. UPGMA (Unweighted Pair Group Method with Arithmetic mean) tree based on Bray-curtis distance matrix of fungal and bacterial sub-sampled OTU (clustered at 97%) table (with even number of sequences in each library, 1036 for fungi and 1558 for bacteria, which counts for 496 OTUs for fungi and 8511 OTUs for bacteria) in Weswood loam soil at 2, 7, and 28 days after amendment with 1% flax SM and 50 µg g^-1^ allyl, benzyl, butyl or phenyl isothiocyanate (ITC). Each OTU count of 3 biological replicates for each treatment were pooled and averaged for calculations on Bray-curtis distance metrics. All sequences were deposited in MG-RAST with accession number of 4515099.3 for fungal ITS and 4515300.3 for 16S. The controls received 1% flax SM but no ITC.


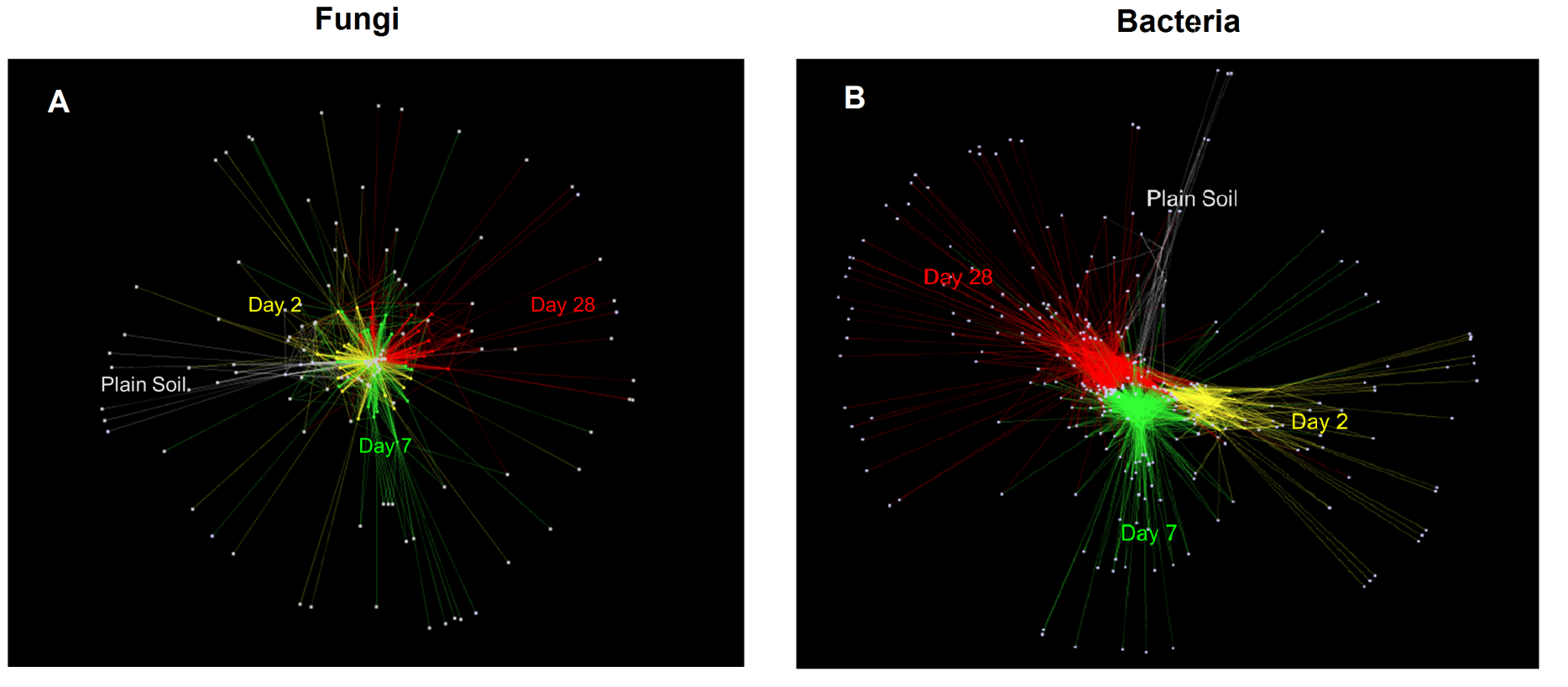


Fig. S2. Soil fungal (A) and bacterial (B) community OTU networks in Weswood loam soil at 2, 7, and 28 days after amendment with 1% flax SM and 50 µg g^-1^ allyl, benzyl, butyl, or phenyl isothiocyanate (ITC), or water as control. The plain soil represents background field soil that received no ITC or flax SM at day 0. All sequences were deposited in MG-RAST with accession number of 4515099.3 for fungal ITS and 4515300.3 for 16S. Analysis was conducted in Cytoscape 2.8.3 using embedded spring algorithm based on operational taxonomic units (OTUs) clustered at 97% sequence identities. Nodes represent OTUs that repelled each other, while edges represent the abundance of shared OTUs among samples. Different amendments have been colored/ grouped by time of incubation.

Table S1. Selected characteristics of Weswood loam soil.

| Soil nutrient concentrations | | | | | | | | | | | | | Soil texture | | | |
| --- | --- | --- | --- | --- | --- | --- | --- | --- | --- | --- | --- | --- | --- | --- | --- | --- |
| Total N | Total C | Organic C | P | K | Ca | Mg | S | Na | Fe | Zn | Mn | Cu | Sand | Silt | Clay | pH |
| ------g kg^-1^ ------ | | | ------------------------------mg kg^-1^ ------------------------------ | | | | | | | | | | --------- %---------- | | |  |
| 0.5 | 12.8 | 5.4 | 34 | 225 | 5440 | 236 | 15 | 210 | 0.95 | 0.15 | 0.61 | 0.26 | 42 | 38 | 20 | 7.9 |

Adapted from Wang et al. 2012

Table S2. Selected elemental and glucosinolate (GLS) concentrations of flax SM.

| **Oilseed** | **C** | **N** | **P** | **K** | **Ca** | **S** | **Mg** | **Na** | **Zn** | **Fe** | **Cu** | **Mn** | **B** | **GLS** |
| --- | --- | --- | --- | --- | --- | --- | --- | --- | --- | --- | --- | --- | --- | --- |
| **Meal** | **------------------------ g kg^-1^ ----------------------** | | | | | | | | **--------- mg kg^-1^ -----** | | | | | **μmol g^-1^** |
| Flax | 491 | 51 | 6.8 | 10.3 | 3.0 | 3.3 | 5.1 | 0.7 | 38 | 33 | 13 | 34 | 27 | N/A |

Adapted from Hu et al. 2011

Table S3.Analysis of similarity based on OTU composition with respect to isothiocyanate (ITC) amendment and time.

| Microbial community | Treatment | ANOSIM metric | |
| --- | --- | --- | --- |
|  |  | *R* | *P* value |
| Fungi | ITC type | 0.2807 | 0.0002 |
|  | Time | 0.5218 | 0.0001 |
| Bacteria | ITC type | 0.2040 | 0.0062 |
|  | Time | 0.9235 | 0.0001 |

Table S4.Analysis of similarity based on taxonomic composition with respect to isothiocyanate (ITC) amendment and time.

| Microbial community | Treatment | ANOSIM metric | |
| --- | --- | --- | --- |
|  |  | *R* | *P* value |
| Fungi | ITC type | 0.2830 | 0.0002 |
|  | Time | 0.5226 | 0.0001 |
| Bacteria (Firmicutes) | ITC type | 0.0721 | 0.1353 |
|  | Time | 0.6379 | 0.0001 |

Table S5. Fungal and bacterial community diversity indexes based upon operational taxonomic units (OTUs; 97% similarity) and their relative abundances in a Weswood loam soil mixed with 1% flax SM and treated with 50 µg g^-1^ allyl, benzyl, phenyl, or butyl isothiocyanate (ITC) and the control receiving no ITC after 2, 7, and 28 days of incubation at 25°C. Diversity and richness estimates were based on reduced sized sequence libraries (1036 sequences for fungal and 1558 sequences for bacterial community respectively). Values displayed represent the mean of 3 biological replicates for each treatment. All sequences were deposited in MG-RAST with accession number of 4515099.3 for fungal ITS and 4515300.3 for 16S.

| Sample | | Community characteristics (mean ± std) | | | | | | | |
| --- | --- | --- | --- | --- | --- | --- | --- | --- | --- |
|  |  | Fungal communities | | | | Bacterial communities | | | |
| Treatment | Day | Observed OTUs | Chao I Richness | Shannon  (H’) | Inverse Simpson | Observed OTUs | Chao I Richness | Shannon  (H’) | Inverse Simpson |
| Control | 2 | 52± 9 | 69±15 | 2.40±0.11 | 6.5±0.5 | 366± 39 | 658±202 | 4.81±0.03 | 47± 9 |
| Allyl | 2 | 61± 5 | 86± 9 | 2.55±0.12 | 7.4±1.7 | 334±141 | 575±185 | 4.28±1.40 | 46±35 |
| Benzyl | 2 | 48± 6 | 75±15 | 2.09±0.09 | 4.4±0.2 | 452± 68 | 837±360 | 5.21±0.11 | 78± 3 |
| Phenyl | 2 | 55± 2 | 76±10 | 2.15±0.14 | 4.8±1.5 | 392± 51 | 645±114 | 4.99±0.18 | 61± 9 |
| Butyl | 2 | 46±14 | 57±13 | 2.12±0.65 | 5.5±3.0 | 455± 29 | 901±159 | 5.20±0.12 | 77±19 |
| Control | 7 | 53± 6 | 79±25 | 1.91±0.15 | 3.3±0.7 | 469± 13 | 992± 45 | 5.23±0.02 | 76± 2 |
| Allyl | 7 | 51± 6 | 82± 7 | 2.08±0.04 | 5.4±0.7 | 494± 44 | 1091±120 | 5.29±0.20 | 82±26 |
| Benzyl | 7 | 52± 9 | 73±10 | 2.05±0.21 | 4.1±0.5 | 504± 27 | 1055± 97 | 5.40±0.21 | 106±37 |
| Phenyl | 7 | 56±10 | 77± 7 | 2.02±0.41 | 3.8±1.5 | 459± 19 | 785± 10 | 5.24±0.17 | 76±21 |
| Butyl | 7 | 44± 6 | 58± 3 | 1.59±0.45 | 2.9±1.4 | 554± 8 | 1005± 96 | 5.65±0.11 | 133±36 |
| Control | 28 | 49± 7 | 90±12 | 1.56±0.09 | 2.5±0.1 | 607± 14 | 1401± 9 | 5.79±0.08 | 183±28 |
| Allyl | 28 | 42±10 | 61±18 | 2.06±0.24 | 5.1±1.0 | 627± 12 | 1090±112 | 5.91±0.09 | 214±44 |
| Benzyl | 28 | 38± 2 | 48± 5 | 1.56±0.14 | 2.7±0.4 | 585± 60 | 1171±186 | 5.76±0.16 | 171±43 |
| Phenyl | 28 | 40± 2 | 56± 8 | 1.70±0.10 | 3.1±0.2 | 593± 18 | 1208±232 | 5.78±0.09 | 173±33 |
| Butyl | 28 | 44± 7 | 64±20 | 1.65±0.15 | 2.8±0.4 | 670± 29 | 1072± 89 | 5.99±0.06 | 195±33 |
